# Burnout, Resilience and Self-Esteem in School Teaching University Students

**DOI:** 10.3390/bs12110422

**Published:** 2022-10-30

**Authors:** Antonio Fernández-Castillo, María J. Fernández-Prados

**Affiliations:** 1 Department of Developmental and Educational Psychology, Faculty of Educational Sciences, Campus de Cartuja S/n., University of Granada, 18071 Granada, Spain; 2 Master in Social Education: Research and Professional Development, Faculty of Educational Sciences, Campus de Cartuja S/n., University of Granada, 18071 Granada, Spain

**Keywords:** burnout, self-esteem, university students, resilience

## Abstract

Burnout syndrome seems to involve fatigue that is characterised by loss of motivation, lack of energy, and some apathy as a consequence of continued exposure to stress in demanding performance circumstances. Background: The goal of the present study is to analyse the relationship between burnout in university students with a degree in Teaching and some variables that may be associated with it such as self-esteem, resilience or age. Methods: A total of 1547 graduate students enrolled in the career of Teaching in the Faculty of Educational Sciences of the University of Granada, Spain, participated in the study. Of them, 337 (21.8%) were men, 1195 (77.3%) were women, 14 (0.9%) indicated other gender options, and 1 (0%) did not respond to this item. The mean age of the participants was 20.52. Results: The results show that low levels of self-esteem and resilience, are the variables that best predict the increase in burnout in students of Teaching. Conclusions: Findings are discussed regarding applied implications and the need for future research. Intervention initiatives focused on enhancing personal strengths such as resilience or self-esteem can help students to cope with the stress associated with demanding educational situations and thus reduce the presence of burnout.

## 1. Introduction

Burnout is quite well known and has been studied for a long time in professional contexts [1], including the educational field, both among teachers and students at various educational levels, including higher education [2,3,4].

Burnout syndrome has been conceptualised as a state of physical and emotional exhaustion characterised by a lack of enthusiasm, feelings of frustration, perception of excessive demands, and decreased performance [5]. Some authors [1,5] have proposed that it is made up of three dimensions: (a) exhaustion and decreased performance energy, (b) the emergence of negative and insensitive feelings towards people with whom the person interacts at work, and (c) low self-evaluation with the consequent decrease in competence and personal commitment.

Other alternative proposals consider burnout as a one-dimensional construct, although they coincide in describing it as a state of emotional, physical and cognitive fatigue that is the result of continued exposure to stress [6,7].

Burnout seems to affect professions that are overloaded and stressful, especially those of a marked social nature or where emotional demands are made, such as caregivers, health workers, social workers and teachers. In these professions, individuals experience distress that, when continued over time, generates burnout [8].

The presence of stress is observed in the educational context, both in teachers, where it is also associated with attempts at dropout, dissatisfaction, loss of interest, lack of involvement, etc., [9] and in students, where it is related to exhaustion, absenteeism, cynicism or poor academic performance, among other negative aspects. In both cases, it is intensified in situations of overload and stress [10,11].

Regarding differences in burnout as a function of age, some studies have found that, at older ages, individuals normally have lower levels of burnout [9], although these studies were not conducted with Teaching students but with other professionals.

Some authors have pointed out that, worldwide, between 40 and 50% of students and health professionals could have burnout to some degree [12]. In fact, it is a phenomenon that can be found in any cultural or geographical context as well as among students of different educational policies and academic degrees [4].

Academic overload, competitiveness and persistent stress in the educational context could drain personal resources, increase dissatisfaction and generate burnout. Furthermore, continued burnout in students has been associated with health problems and even suicidal ideation [12].

In some approaches, burnout has been considered an indicator of a lack of vocation or low resilience, and there seems to be an association between the two concepts [13]. Other researchers have specifically found that higher levels of resilience could be associated with lower levels of burnout in university students [14].

The term resilience has often been understood as an adaptive ability to transform challenges into opportunities, to learn from demanding situations, or, ultimately, to react and face adversity efficiently and recover from it [15].

Resilience could be a psychological skill that would help students to better cope with stress and, therefore, prevent the onset of negative aspects arising from demanding situations in the educational context [16].

Some authors have shown that the basic sources of stress and adverse circumstances in students can be reduced to three: teachers, peers, and parents, with resilience, specifically focusing on each of them [17,18]. These studies give importance to the perceived degree of resilience to adverse events that students confront in their relationships with teachers, peers and parents.

Another variable related to burnout is self-esteem. Some authors have found that the two variables correlate significantly and negatively with each other and that self-esteem may even buffer the negative effects of a stressful demand on burnout [19].

A traditional way of conceptualising self-esteem is to understand it as a global assessment, more or less positive, that individuals make of themselves [20,21]. High levels of self-esteem have been associated with a greater ability to cope with stress in demanding situations [19,22] and, specifically, also with lower levels of burnout [23]. Conversely, low self-esteem has been associated with many negative aspects for the individual, including suicidal ideation [24].

As mentioned, the relationship between self-esteem and burnout seems clear and negative, also in students [25]. A possible explanation for this influence is that self-esteem could have a beneficial effect on some coping strategies that, in turn, would reduce burnout. Thus, higher levels of self-esteem have been associated with less fatigue and higher levels of personal fulfilment [25].

Although there is considerable evidence of the presence of burnout in students and its relationship with aspects such as resilience and self-esteem, there are few studies that have studied this relationship in Teaching students. Future educators must have a clear vocation for a profession in which it is common to find some degree of burnout.

Therefore, in this study, we set the following objectives. The first goal is to explore the presence of burnout in Teaching students and the possible differences between men and women. The second focuses on studying the possible association between the presence of burnout and some target variables, such as self-esteem, three of the main manifestations of resilience (professors, peers and parents) in students and students’ age. Thirdly, we proposed to analyse possible differences in burnout depending on students’ age and gender. Finally, the fourth objective aimed to explore whether the target variables (self-esteem and general resilience) can predict the onset of burnout in Teaching students and if so, to what extent.

The main hypotheses of our study can be summarised as follows: First of all, we expect to find around 30% of students with high levels of burnout, according to the studies reviewed [4,12]. Secondly, and in line with what was found in other studies [14,25], we expect to find an association between the presence of burnout and the following variables: self-esteem, resilience and age. Specifically, following what was found in previous studies [9,14,25], we expect to find (a) that older students have lower levels of burnout; (b) higher self-esteem score leads to lower burnout score and (c) resilience has a negative effect on burnout in university teaching students.

## 2. Materials and Methods

### 2.1. Participants

In this investigation, participants were 1547 graduate students enrolled in the career of Teaching with specialties in Early Childhood Education (631 (40.8%)) and Primary Education (916 (59.2%)) in the Faculty of Educational Sciences of the University of Granada, Spain.

**Figure 1:**
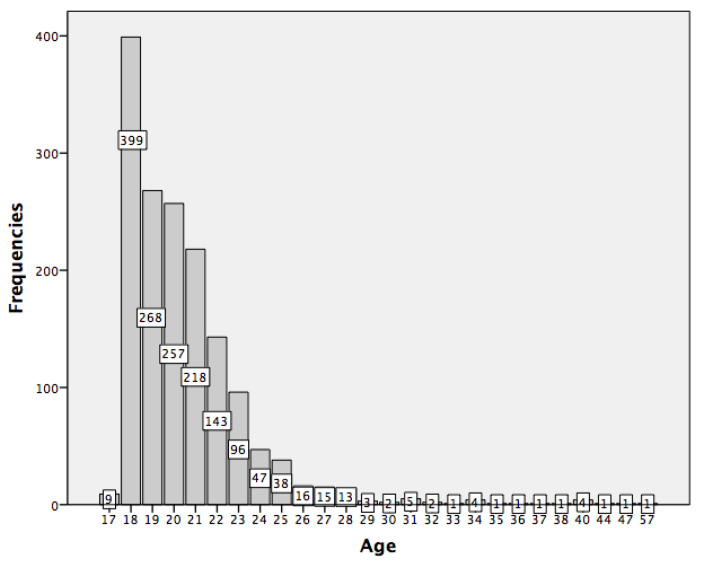
Age frequency distribution in the sample.

The participants’ age was between 17 and 57 (M = 20.52, SD = 3.08). The frequency distribution is shown in Figure 1. Regarding gender, 337 (21.8%) were men, 1195 (77.3%) were women, 14 (0.9%) indicated other options and 1 (0%) did not respond to this item.

Of them, 758 (49%) were first-year students, of whom 400 (25.9%) were evaluated in the first month of the beginning of the career, and 358 (23.1%) at the end of the first course, 334 (21.6%) were second-year students, 305 (19.7%) were third-year students, and 150 (9.7%) were fourth-year students.

Inclusion criteria were voluntary participation in the study and to be enrolled in a Teaching career.

### 2.2. Measures

For the assessment of burnout in the participants, the Spanish population-validated version [11] of the One-Dimensional Student Burnout Scale [7,26] was used. The scale is made up of 15 items with four response options ranging from 1 (never) to 4 (always), with the total scores ranging between 15 and 60 and a midpoint of 37.5. Some examples of items in the questionnaire are: “Having to attend classes daily makes me tired” or “My school problems make me depressed easily”. Regarding its psychometric properties, in previous studies [7], Cronbach’s alpha of 0.91 was reported, while the split-half reliability, according to the Spearman–Brown formula, was 0.89. In the Spanish population adaptation study, Cronbach’s alpha reached a value of 0.90 [11]. For this study, Cronbach’s alpha value was also 0.90.

To assess self-esteem, the Spanish population adaptation [27] of the Rosenberg Self-Esteem Scale [21] was used. The instrument is structured in 10 items (half of them are score-reversed because they are worded inversely and negatively) that are rated from 1 (strongly disagree) to 4 (strongly agree), providing a general self-esteem score ranging from 10 to 40. In the study of the Spanish version of the scale [27], Cronbach’s alpha values between 0.85 and 0.88 were obtained. In the present study, it reached the value of 0.83.

To assess resilience, the Subjective Resilience Questionnaire (SRQ) [17,18] was used. The questionnaire provides a general assessment of resilience as well as three specific indicators or dimensions concerning peers, teachers, and parents. It presents 30 items of which half are expressed negatively. Each of the three dimensions has 10 items. Examples of instrument items can be: *“Sometimes my friends criticize me for not doing something well instead of trying to help me, but that doesn’t decrease my effort to improve myself”, “Though sometimes I’m not appreciated by my teachers due to my limitations and errors, in these occasions I do not discouraged, and go on trying to learn”, “If my parents ignore me when I need them to help me with a problem, I get discouraged and stop striving to solve it”*. In the work of Alonso-Tapia et al. [17], a reliability index for the general scale of 0.97 was reported: 0.98 for the Teacher subscale, 0.93 for the Peer subscale 0.93, and for the Family subscale. In our study, Cronbach’s alpha for the general scale was 0.91, for the Teacher subscale α = 0.79, for the Peer subscale α = 0.77, and for the Family subscale α = 0.77.

Participants were also informed about their gender and age.

### 2.3. Procedure

To collect the data, first, we contacted the teaching staff that teaches the different courses and groups of the target careers at the Faculty of Educational Sciences of the University of Granada. Permission was granted to access their classes to evaluate the students. The investigators briefly informed the students about the goals of the investigation, emphasising that participation was voluntary and anonymous, that no benefit or remuneration would be obtained for participating, and that they could leave the classroom at any moment if they wanted to. We asked them to respond to all the items.

After the students had completed the assessment instrument, the data were entered into the database created for this research.

### 2.4. Data Analysis

To analyse the data, firstly, we conducted descriptive and frequency analyses. Secondly, we performed Pearson’s correlational analysis, multiple linear regression analysis and comparison of means. The level of significance for all analyses was *p* < 0.05. The analyses were performed using the Spanish version of the statistical program SPSS, version 24.

## 3. Results

For our first objective, we carried out a basic descriptive analysis of burnout. The mean and standard deviation of burnout and the other target variables are shown in Table 1.

**Figure 2:**
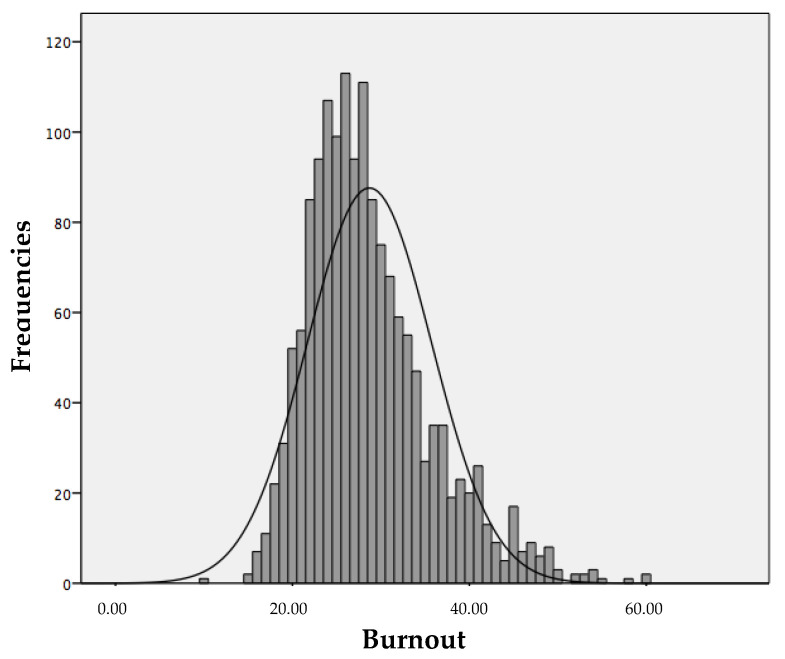
Distribution of burnout frequencies in the sample.

Our results for burnout show a distribution that accumulates a large number of cases below the central point of the distribution (37.5), with the mean = 28.71. Only 176 subjects (11.4%) scored above the central point, whereas 1371 (88.6%) scored below. To describe the proportion of students according to their level of burnout, four cut-off points were established in the distribution of possible scores, following the criteria of the original studies of the scale [28]. In this way, four groups of subjects were obtained based on their score on the general burnout scale (no burnout (scores between 15 and 26.25): *n* = 680, 44%; mild level (scores between 26.25 and 37.50): *n* = 691, 44.7%; moderate (scores between 37.50 and 48.75): *n* = 154, 10%; and profound (scores between 48.75 and 60): *n* = 22, 1.3%). The distribution of general burnout is shown in Figure 2.

The second of our objectives was to explore the possible association between burnout and several variables. Pearson’s correlational analysis not only included the level of self-esteem and the three target indicators of specific resilience, but also the participants’ age. The results can be seen in Table 2.

The results show a significant correlation between the variables. Thus, a lower level of general burnout was significantly associated with higher levels of self-esteem and resilience in all of the target variables, and students’ age.

**Figure 3:**
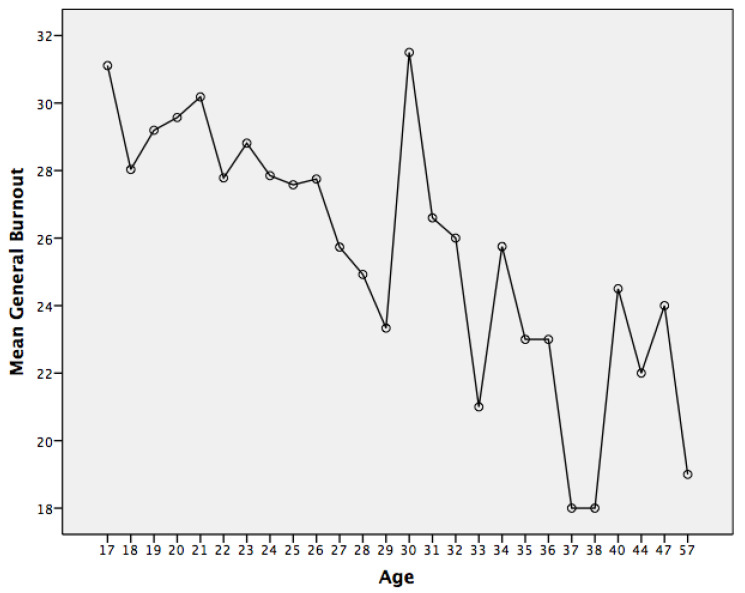
Mean general burnout as a function of age.

For the third of our objectives, determining differences in burnout as a function of students’ age, the ANOVA (obtained in the mean comparison test, where burnout was taken as the dependent variable and age as the independent variable) showed a significant relationship, F(25, 1521) = 1.85, *p* = 0.007, indicating that at higher ages, students show lower levels of burnout, as can be seen in Figure 3. To know the value of the power of the effect, the Eta Squared test was carried out. The obtained value was 0.03, which indicates a low effect size.

As regards differences between men and women in the main variables of the study, non-parametric tests were carried out. The results, which are detailed in Table 3, indicate higher levels of resilience and self-esteem in men than in women. No significant differences in burnout were found between men and women.

The fourth objective was to explore whether the target variables (self-esteem and general) can predict the onset of burnout in Teaching students and if so, to what extent. For this, a multiple regression analysis was carried out. The results are detailed in Table 4.

**Figure 4:**
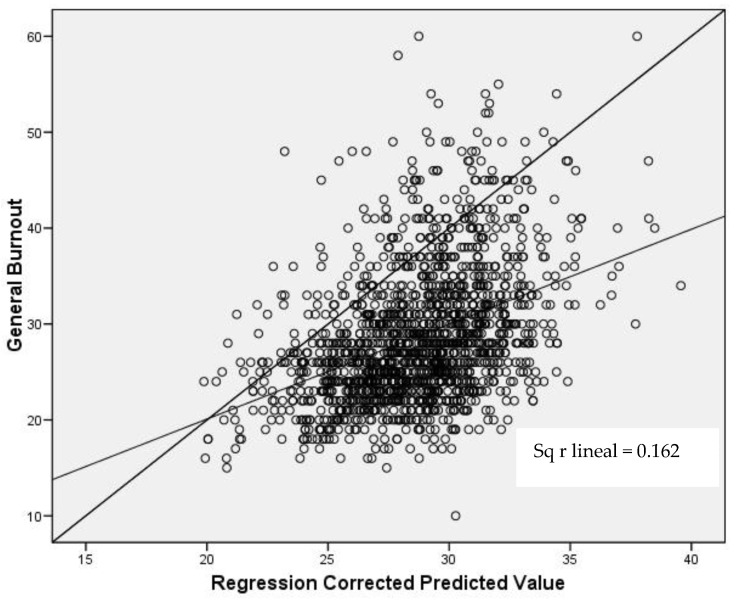
Scatter plot of the model.

According to the adjusted R^2^, the model explained 17% of the variability of burnout. The regression equation (Y = a + b × X1 + b × X2) can be expressed: [Burnout = 50.37 + (−0.29 × SE) + (−0.12 × R)]. As can be seen in Table 3, the variance inflation factor (VIF) indicates the absence of multicollinearity in the model variables. As can also be seen in the scatter plot (Figure 4), the model fit is Good. The results showed that resilience and self-esteem were good predictors of burnout in the participating students, with general resilience as the best one.

## 4. Discussion

One of the objectives of this work was to descriptively explore the presence of burnout in the sample. The mean reached a relatively low value (M = 28.71, SD = 7.04) considering the range of scores, between 15 and 60. The results do not confirm our working hypothesis and lead us to conclude that most of the participants in the sample did not achieve high or very high burnout scores. This result contrasts with other studies that had shown burnout percentage values around 40 or 50 [12]. However, these studies focused on students of health specialties such as Medicine, not on teaching students.

The correlation analyses carried out to test our second objective showed that all the analysed variables had a significant and negative association with burnout, both in the global sample and in the subsamples of men and women. High levels of self-esteem, resilience in the specific dimensions (professors, peers and parents), and higher age are associated with lower levels of burnout. This result confirms our working hypothesis is in line with studies that consider that self-esteem and resilience usually present this association in other populations [13,14,23]. Age was specifically studied in our third objective. Some authors [9] pointed out that higher age is associated with lower levels of burnout, and this is also the trend we found in our data.

Higher age could imply greater coping skills with aspects inherent to stress and overload, which have been associated with the onset of burnout. In any case, this result should, that confirms our working hypothesis, be considered with caution because the range of ages considered and the dispersion of this variable in our sample are not very broad. In addition, the result of the power of the effect test found is relatively low. Therefore, future studies could examine the implication of age and maturation in the onset and development of burnout in students of educational sciences. Regarding the differences according to sex, our results must be taken with caution given the imbalance in the composition of our sample, where there is a significant majority of women. It would be a good idea to replicate these analyses in future studies with a similar population of students.

Finally, the contrast of our fourth objective revealed that the two considered variables, self-esteem and general resilience (that included coping with professors, parents and peers), are good predictors of reduction in burnout, with resilience being the one with the greatest predictive power. This result underlines the importance of self-esteem and resilience in coping with stress and overload in the educational context, confirms our working hypothesis and is consistent with prior evidence [13,14,23]. In this sense [16], resilience could be helpful to cope with stress and prevent burnout concerning professors, parents and peers.

Self-esteem is also another relevant variable due to its role in reducing burnout. Self-esteem may strengthen some coping strategies that, in turn, would reduce stress, helping to reduce exhaustion and increase personal fulfilment. [25]. A more advanced analysis could consider the interaction between resilience and self-esteem and its influence on burnout. This objective could also be of interest in future studies.

Taking into account the interest of university institutions in reducing student dropout and failure, as well as promoting their success and well-being [29], the importance of determining which aspects affect burnout is clear. This would allow for the design of effective intervention initiatives to reduce stress levels and help students cope with the demands of certain tasks associated with the onset of burnout. Examples of this are specific interventions focused on enhancing resilience or those based on Mindfulness, among other personal resources, due to their association with lower levels of burnout [29,30].

It would be very interesting to work on resilience and stress-coping strategies in Teaching students due to their effectiveness in reducing burnout and enhancing engagement [31,32]. Especially in future professionals of a profession of a marked social nature, in which, due to its emotional demands, some stress is usually experienced, especially, for being a profession with high social relevance.

## Figures and Tables

**Table 1:** Means and standard deviations of the variables in the general sample.

Variable	Mean	SD
Burnout	28.71	7.04
Self-Esteem	31.40	4.58
General Resilience	99.79	16.64
Resilience concerning Professors	33.33	6.24
Resilience concerning Peers	33.16	6.08
Resilience concerning Parents	33.29	6.30

**Table 2:** Correlations between the target variables and burnout.

	Burnout General Sample	Burnout Men	Burnout Women
Self-Esteem	−0.30 **	−0.40 **	−0.28 **
Resilience concerning Professors	−0.38 **	−0.44 **	−0.36 **
Resilience concerning Peers	−0.33 **	−0.37 **	−0.31 **
Resilience concerning Parents	−0.28 **	−0.37 **	−0.25 **
Students’ Age	−0.08 **	−0.10 **	−0.08 **

** *p* < 0.01.

**Table 3:** Gender differences in the main variables.

Variable	Gender	Average Range	Mann–Whitney Test
Burnout	Male Female	763.53 767.34	U = 200,357.50; *p* = 0.80
General Resilience	Male Female	815.04 752.81	U = 184,998.00; *p* = 0.02
Self-Esteem	Male Female	833.59 747.00	U = 178,050.00; *p* = 0.00

**Table 4:** Multiple regression analysis: Burnout in students and some target variables.

Criterion	Predictors	β Standardised	*p*	VIF
Burnout	General Self-esteem (SE) General resilience (R)	−0.19 −0.30	0.00 0.00	1.16 1.16

F(2, 1543) = 153.084, *p* < 0.00. *R* = 0.41; *R*^2^ = 0.17; Adjusted *R*^2^ = 0.16; Error = 6.44.

## Data Availability

The data and materials are available from the corresponding author upon reasonable request.
